# A Phase Transition of the Unconscious: Automated Text Analysis of Dreams in Psychoanalytic Psychotherapy

**DOI:** 10.3389/fpsyg.2020.01667

**Published:** 2020-08-12

**Authors:** Alessandro Gennaro, Sylvia Kipp, Kathrin Viol, Giulio de Felice, Silvia Andreassi, Wolfgang Aichhorn, Sergio Salvatore, Günter Schiepek

**Affiliations:** ^1^Department of Dynamic and Clinical Psychology, Sapienza University of Rome, Rome, Italy; ^2^Institute of Health and Social Relations, FOM, University of Applied Sciences for Economics and Management, Essen, Germany; ^3^Institute of Synergetics and Psychotherapy Research, University Hospital of Psychiatry, Psychotherapy and Psychosomatics, Paracelsus Medical University, Salzburg, Austria; ^4^Faculty of Psychology, NCIUL University, London, United Kingdom; ^5^Department of Psychology, Ludwig Maximilian University, Munich, Germany

**Keywords:** text analysis, dream analysis, psychotherapy process, meaning, phase transition

## Abstract

**Aim:**

Psychotherapy could be interpreted as a self-organizing process which reveals discontinuous pattern transitions (so-called phase transitions). Whereas this was shown in the conscious process of awake patients by different measures and at different time scales, dreams came very seldom into the focus of investigation. The present work tests the hypothesis that, by dreaming, the patient gets progressively more access to affective-laden (i.e., emotionally charged) unconscious dimensions. Furthermore, the study investigates if, over the course of psychotherapy, a discontinuous phase transition occurs in the patient’s capacity to get in contact with those unconscious dimensions.

**Methods and Procedures:**

A series of 95 dream narratives reported during a psychoanalytic psychotherapy of a female patient (published as the “dreams of Amalie X”) was used for analysis. An automated text analysis procedure based on multiple correspondence analysis was applied to the textual corpus of the dreams, highlighting a 10-factor structure. The factors, interpreted as affective-laden unconscious meaning dimensions, were adopted to define a 10-dimensional phase space, in which the ability of a dream to be associated with one or more local factors representing complex affective-laden meanings is measured by the Euclidean distance (ED) from the origin of this hyperspace. The obtained ED time series has been fitted by an autoregressive integrated moving average (ARIMA) model and by non linear methods like dynamic complexity, recurrence plot, and time frequency distribution. Change point analysis was applied to these non linear methods.

**Results:**

The results show an increased frequency and intensity of dreams to get access to affective-laden meanings. Non linear methods identified a phase transition-like jump of the ED dynamics onto a higher complexity level of the dreaming process, suggesting a non linear process in the patient’s capacity to get in contact with unconscious dimensions.

**Conclusion:**

The study corroborates the hypothesis that, by dreaming, the patient gets progressively more access to affective-laden meaning intended as unconscious dimensions. The trajectory of this process has been reproduced by an ARIMA model, and beyond this, non linear methods of time series analysis allowed the identification of a phase transition in the unconscious process of the psychoanalytic therapy under investigation.

## Introduction

Process research is an essential source of knowledge on how psychotherapy works. Especially in psychoanalysis and in psychodynamic psychotherapies, single-case reports and case studies on change dynamics have a long tradition of more than one century, founded by Sigmund Freud. There is a great variety of case studies, from qualitative reports to sophisticated time series analysis studies. Reviews on the methodology of single-case research in psychodynamic psychotherapy were published by Hilliard (1993) and Fonagy and Moran (1993). Beyond the frame of psychoanalysis, the non linear dynamic systems approach promoted the field of case-related research designs by using non linear methods in order to understand chaotic dynamics and self-organized pattern transitions of cognitive, affective, and interpersonal processes (Kowalik et al., 1997; Schiepek et al., 1997, 2016a,b, 2018). Gumz et al. (2012) connected both fields by applying the method of dynamic complexity (DC) (Haken and Schiepek, 2010; Schiepek and Strunk, 2010) on the process ratings of patients and therapists in psychodynamic psychotherapies.

Usually, process research using high-frequency measures is based on transcripts, video tapes, socio-physiological measures, continuous self-reports, or electronic diaries. In most of the studies, the focus is on intra-session dynamics, with advanced developments in inter-session dynamics by using ecological momentary assessment or internet-based real-time monitoring (Schiepek et al., 2016a, b; 2018; Steffensen et al., in review). Most of the applied measures were taken from awake subjects during the therapeutic interaction or during moments of triggered self-reflection (e.g., writing diaries or doing quantitative self-assessments). Only few studies focused on dreams which were reported during the psychotherapeutic process.

One of the pioneering steps to relate changes of dream patterns to therapeutic change and personality development was done by Roesler (2018a, b). He used the qualitative method of structural dream analysis (SDA) in order to identify dream patterns and connected them to the psychological problems of the dreamers and to psychotherapeutic changes and to their personality development. SDA allows a systematic analysis of dream texts and contents during the therapeutic process. Older studies showed that the themes of dreams change during psychotherapy (Cartwright, 1977) and that dream themes correspond to the session protocols reflecting the current conflictual themes in the waking life of the dreamer (Greenberg and Perlman, 1978). Popp et al. (1990) compared the narratives of dreams and of therapy sessions using the method of Core Conflictual Relationship Themes (CCRT), showing that both narratives were structured by the same unconscious relationship patterns. Glucksman and Kramer (2004, 2006, 2012) highlighted a significant correlation between clinical improvement and thematic changes from the initial to the final dreams of the treatment. Qualitative therapeutic changes of recurrent post-traumatic nightmares were observed after focusing-oriented dream work (Ellis, 2016). Widmer (2019) applied the SDA method to a dream series which was documented by Kächele et al. (1999; Thomä and Kächele, 2006; “Amalie’s Dreams,” see “Methods”) and found increased ego strength following five different dream patterns proposed by Roesler (2018a).

In summary, literature suggests that dreams are able to mirror clinical progress and personality development. Dreams prepare and pre-develop new ways of organizing life experiences and new ways of the patient’s mental functioning. In accordance with Jung (1971), by dreaming, the unconscious brings new information to the conscious processing of a self-regulating system—the “psyche.” By dreaming, the patients get access to increasingly deep affective-laden unconscious meanings on which the patient’s relational and emotional life is grounded (see Venuleo et al., 2018). Following this pathway, changes and pattern transitions should occur in the contents and meanings of dream narratives during psychotherapy. The aim of the present study is to investigate the temporal evolution of the unconscious dimensions lying in the dreams of a single case of psychoanalysis.

### Dreams and Change Processes

Dreams create or construct meaning in a way which is not restricted to the rules of conscious everyday thinking (secondary processes). The mental processes of dreaming are usually more affectively charged, and their functioning lies beyond the restrictions of time, space, causality, physical laws, and logic (primary processes). The unconscious is the mind’s basic way of functioning, but it is also related to rational thinking. This relationship is framed by the concept of *affective semiosis* (Salvatore and Freda, 2011), which describes the functioning of minds by predominant primary processes but also by the activity of functions of differentiation (secondary processes). The affective and pre-semantic activity of interpreting experiences orients the processes of feeling and thinking. The affective semiosis frames and orients rather than executes regulative functions and provides the context as an implicit embodied presupposition which orients subsequent perceptive and cognitive activities. This relational context can be seen as the preferential trajectory of semiotic intersubjective activity of producing and interpreting signs. The dynamic relational context promotes the flow of signs, and the flow of signs (re)produces the context. With reference to Matte Blanco’s (1975) “bi-logic approach” and Ciompi’s (1982) “affective logic,” in the process of affective semiosis, thoughts emerge from emotions, and it shapes the flow of experiences into a contextual field conveying affective meanings.

Psychotherapy is an intersubjective process (Salvatore et al., 2010; Gennaro et al., 2011, 2017; Salvatore and Gennaro, 2012) which creates changes in self-concepts, schemata, or ways of problem solving (Nitti et al., 2010; Salvatore et al., 2010). In such process, as pointed out by Moser and von Zeppelin (1996), the organization of dreams may be considered as an entangled set of affective-cognitive procedures generating a micro-world—the dream—by which a person attempts to find a solution for an activated conflict. In this unconscious process of problem solving, information processing integrates cognitions and emotions (affective-laden thoughts). Accordingly, it may be expected that dreams produce contexts which allow us to get access to affective-laden meaning dimensions (i.e., unconscious) and to test new meanings as organizers of the patient’s internal and external world.

The emergence of new meanings is a dialogical and contextual process which creates new narratives of dreams in the clinical setting. Specifically, as highlighted by the two-stage semiotic model (TSSM) (Salvatore et al., 2010), good outcome psychotherapies are characterized by alternating phases of consolidation and innovation of meanings (Gennaro et al., 2010, 2017; Salvatore et al., 2010; Rocco et al., 2018). This alternation is nourished by the recursive interplay between pre-semantic affective meanings underpinning the patient’s sensemaking and their cognitive elaboration. Accordingly, the progress of psychotherapy is driven by allowing the patient to get access to increasingly deeper affective dimensions and shaping his life experiences and therefore facilitating the emergence of new meanings and their cognitive elaboration enriching a patient’s ability to access increasingly deeper affective dimensions and their cognitive elaboration (Mergenthaler, 1996; Mergenthaler and Bucci, 1999; Bucci, 2011; Gennaro et al., 2011, 2017; Rocco et al., 2018).

In line with synergetics (Haken, 2004; Haken and Schiepek, 2010; Schiepek et al., 2016a), sensemaking could be described as a cognitive, affective, and intersubjective process in which emerging patterns (called “order parameters”) enslave the mental states of both patient and therapist. In consequence, the narratives of the dreams, the experienced scenes of the dreams, and the interactive process of psychotherapy will be synchronized. Order parameters emerge from the interaction of many parts of a system and can be seen as constitutive patterns of the field dynamics. Emerging patterns result from phase transitions which represent qualitative changes of a system’s modalities of working. Patterns of clinical change and transitions of cognitive-affective modes during psychotherapeutic processes have been analyzed and modeled in previous studies (e.g., Lauro-Grotto et al., 2009; Salvatore et al., 2009; Haken and Schiepek, 2010; de Felice and Andreassi, 2014; Schiepek et al., 2014, 2016a,b; Halfon et al., 2016; de Felice et al., 2019; Olthof et al., 2019; Schiepek et al., in press; Walter et al., 2010) and were simulated by a theoretical (mathematical) model of psychotherapeutic change (Schiepek et al., 2017; Schöller et al., 2018). Furthermore, the research based on the TSSM (for a review, see Gennaro et al., 2010; Nitti et al., 2010; Salvatore et al., 2010; Salvatore and Gennaro, 2015) highlights how specific patterns characterize the course of good outcome psychotherapies.

According to this framework, we hypothesize that through the course of a successful psychotherapy, the dream narratives increase the specificity of their affective charge as a marker of the patient’s capacity to get access to increasingly deep affective-laden dimensions of sensemaking (HP1). Moreover, due to the field dynamics of sensemaking underpinning the clinical exchange, the increased affective charge of the dream narratives should follow a non linear trend (HP2).

Finally, we expect that the self-organizing process of the psychotherapy will show an increase in the complexity of dream dynamics within the phase space of the affective-laden meaning dimension (HP3). The dream dynamics should realize a discontinuous (non-stationary) evolution (phase transition), and a sudden change in complexity should be visible in different complexity measures.

## Materials and Methods

### The Patient and the Dream Texts

Ninety-five dream narratives reported by a female patient during 517 psychoanalytic sessions (Kächele et al., 1999) were subjected to a procedure of text analysis (see below). Verbatim transcripts of the dreams were available from the “Inventory of Amalie’s Dreams” at the University of Zürich (Meissner et al., 2009). Given that this is published material for scientific purposes, no ethics approval was needed. The full-text corpus of all 95 dreams had a length of 38,733 words with a range between 29 words for the shortest and 1,497 for the longest dream reports.

The female patient known under the pseudonym “Amalie X” was diagnosed with dysthymia (F34.1 in the ICD-10, which corresponds to chronic depression) and with disorder of sexual identity (F64). Since her adolescence, she has suffered from chronic hirsutism—an excessive hair growth on parts of the body where hair is normally absent or minimal. The hirsutism caused psychological distress and social difficulties, avoidance of social situations, and symptoms of anxiety and depression. On the other hand, distress intensified the abnormal hair growth, and this was the reason that she self-referred to psychotherapy in addition to endocrine therapy. The patient suffered from a reduced self-esteem and a distorted body image and challenged her sexual identity as a woman. In consequence, she avoided any closer, especially sexual, contacts with men. The details of the psychoanalytic therapy which was realized by a session frequency of about three times per week are reported in Kächele et al. (1999). Different outcome and process measures [e.g., self-esteem (Neudert et al., 1987) or psychological strain (Neudert and Hohage, 1988)] considered the case as good outcome (see also Thomä and Kächele, 2006).

### Dreams Text Analysis

We performed, by means of T-Lab software (Lancia, 2002, 2005), an automated procedure of text analysis over the transcripts of Amalie’s dreams. This analysis is an Automated Co-occurrence Analysis for Semantic Mapping (ACASM) adjusted for the specific dataset (for specific details of the method and its rationale, see Gennaro et al., 2010; Salvatore et al., 2012, 2017a,b). ACASM was developed in order to identify thematic contents in texts by a cluster analysis procedure applied to words which are associated with each other. The procedure of text analysis we used in this study overlaps the ACASM procedure in the algorithms of text segmentation and in the identification and selection of the lexical forms under analysis. Unlike the ACASM procedure, which performs a cluster analysis procedure to get a semantic description of a text, the present works adopted a correspondence analysis (see below).

The text analysis procedure was performed according to the following steps. Firstly, the textual corpus of dream narratives was split into units of analysis, called elementary context units (ECUs). Each ECU corresponds to a dream narrative. Secondly, the lexical forms present in the ECUs have been identified and categorized according to the “lemma” they belong to. A lemma is the citation form (namely, the headword) used in a language dictionary: for example, word forms such as “go,” “goes,” “going,” and “went” have “go” as their lemma; “child” and “children” have “child” as their lemma. Thirdly, lemmas were ranked according to their frequency. The 5% highest-frequency lemmas were omitted by the fact that the higher the frequency of a lemma, the less it contributes to the discrimination among the ECUs. High-frequency lemmas (e.g., words like “and,” “to,” and “of”) are not specific to any ECU. Then the lemmas were ranked according to their frequency, and the 10% most frequent lemmas were selected in order to obtain a digital matrix of the corpus, having as rows the ECU (i.e., the dream narratives), as columns the lemmas, and in the cell x*_*ij*_* the value “1” if the *j*th lemma was contained in the *i*th ECU; otherwise, the x*_*ij*_* cell received the value “0.” A correspondence analysis on the obtained matrix allowed retrieval of the factors describing lemmas having higher degrees of association, i.e., occurring together many times. According to Salvatore et al. (2015, 2017a, 2017b), each factor was interpreted as an affective-laden meaning characterized by strongly associated (co-occurring) lemmas (see the Appendix of this article).

Based on the factors, a multidimensional phase space was created in which each dream is represented in terms of a vector whose components are described in terms of the squared cosine, representing the correlation between a dream and a factor. The higher the squared cosine of a dream for a specific factor, the higher the fitting of a dream with the respective affective meaning (factor).

### Affective Charge Analysis

For each dream vector, the Euclidean distance (ED) from the origin of the factor space has been computed. The higher the ED value, the greater the *local* incidence of one or more factors representing a dream in the phase space. The ED has been interpreted as the degree to which a certain dream is able to reach affective-laden meanings described by the factors. The higher the ED value, the greater the incidence of one or more factors determining the meaning of a dream. The incidence of polarized values on factors has been considered as a marker of the affective characteristics of the meaning (Salvatore and Freda, 2011; Salvatore et al., 2017a, b). The arithmetic average of all ED vectors was calculated in order to define a *centroid* around the origin of the phase space. By this, each dream vector was categorized as lying inside or outside of the centroid. Dreams categorized as lying outside of the centroid, that is, having an ED in the multidimensional factor phase space greater than the average of the EDs of all dreams, could be addressed as highly associated with one or more factors; in other words, they address the affective-laden meanings displayed by the factors.

### Time Series Analysis

In order to test the hypothesis of an increased frequency of getting access to affective-laden meanings during psychotherapy (HP1), a logistic binary regression model was calculated with “time” (the sequence of dreams from 1 to 95) as the independent variable and the categorization of dreams as lying inside or outside of the centroid as the dependent variable.

In order to test the extent by which affective-laden meanings are reached (HP2), a non linear model of the process has been tested. Specifically, we adopted an autoregressive integrated moving average [ARIMA_(_*_*p*_*,*_*d*_*,*_*q*_*_)_] model^1^. ARIMA_(_*_*p*_*,*_*d*_*,*_*q*_*_)_ is a data-based modeling procedure which derives stepwise predictions and formalizes the variation of a time series as a function of one or more predictors (e.g., the time series itself) and stochastic noise (McCleary and Hay, 1980; Hyndman and Athanasopoulos, 2014; Jebb et al., 2015). In ARIMA_(_*_*p*_*,*_*d*_*,*_*q*_*_)_ models, *p* is the autoregressive component representing a linear regressive dependency of a time series value on its preceding *p* values, *d* indicates the order of differencing that has been applied to the time series in order to remove any trend from the data, and *q* represents a linear regression of a current value of the time series against prior random errors (McCleary and Hay, 1980; Jebb et al., 2015). In our case, we adopted an ARIMA_(1_,_1_,_1)_ model (see section “Results”) with “time” (the sequence of dreams from 1 to 95) as an independent variable and the EDs as a dependent variable.

### DC Analysis

According to the third hypothesis, the EDs of each dream were subjected to three procedures of complexity analysis: DC, recurrence plot (RP), and time frequency distribution (TFD).

DC (Haken and Schiepek, 2010; Schiepek and Strunk, 2010) is the multiplicative product of a fluctuation measure and a distribution measure applied to discrete time series (in this case, the ED sequence of all dreams) with a given data range [*x*_*min*_, *x*_*max*_]. The fluctuation is sensitive to the amplitudes and frequencies of a time signal, and the distribution scans the scattering of values over the range of possible values. In order to identify non-stationarity, DC is calculated within a moving window running over the time series (window width: 7, overlap: step 1). The window width was chosen as seven because this corresponds to other applications of the DC method on psychotherapy processes and because with this small width, we do not lose too many time points. On the other hand, seven measurement points is sufficient to calculate the DC.

RPs identify recurrent patterns of time series in a time × time diagram (Eckmann et al., 1987; Webber and Zbilut, 1994). Snippets of a full time series are embedded in a phase space with time-delay coordinates. The number of time-delay embedding coordinates corresponds to the snippet length (here: 3), and the time delay *τ* between the embedded measurement points is defined by the first zero-crossing or the first minimum of the autocorrelation of the time series (here: *τ* = 1). By this method, each snippet of the time series is embedded in the time-delay phase space by a vector point. The cell entries in the time × time RP are the EDs between the vector points (distance matrix) which are rainbow color-coded with blue (smallest distance) = recurrent to red (longest distance) = transient. In an RP, recurrent patterns and their transients become apparent.

TFD is a method to calculate and visualize the frequency of a signal (time series) as it changes with time (Cohen, 1989; Sejdić et al., 2009). In order to identify frequency changes, a moving window approach is implemented. Mathematically, both time *t* and frequency ω are variables of a distribution *P*(*t*, ω) which describes the amplitude of the signal at each given *t* and ω. Here, we use the so-called Stockwell transform (*S*-transform), which is a combination of two common TFD methods, the short time Fourier transform and the continuous wavelet transform (Stockwell et al., 1996). It preserves the phase information available from the former method but uses the variable (i.e., not fixed) window length of the continuous wavelet method. For visualization, time and frequency are plotted on a plane (*x*: time, *y*: frequency), and color coding is used for the representation of the amplitudes of the frequencies.

In a last step of testing the non linear phase transition hypothesis, the ED time series, the DC time series, the RP, and the TFD of the ED time series were subjected to a change point analysis (CPA, Killick et al., 2012) in order to identify the time point where a phase transition occurs. CPA is sensitive to changes of specific statistical properties of a time series *x*. It contains a change point if it can be split into two segments *x*_1_ and *x*_2_ such that *C*(*x*_1_) + *C*(*x*_2_) < *C*(*x*), where *C* represents the cost function, *C*(*x*) = *N*var(*x*), and *N* is the number of time points of *x*. In other words, a change point is detected between the segments *x*_1_ and *x*_2_ if the sum of the variance of the statistical property of interest, e.g., the mean of the segments, is smaller than the variance of this property of the whole time series; otherwise, no change point is detected. In our application, we used CPA for the detection of changing variance in the ED time series. The analysis was done with the function *ischange* implemented in Matlab.

## Results

### Preliminary Dream Text Analysis

The procedure of multiple correspondence analysis (MCA) applied to the dreams × lemmas matrix identified 10 factors which explained 27.84% of the variance of the matrix. In light of the high dispersion of the data in the matrix under analysis, this represents a high percentage of explained variance (see Benzecri, 1973, 1979; Bolasco, 1999), especially with respect to the fact that the rate of variance depends on the number of variables under analysis—280 lemmas in our case.

### Hypothesis 1

**FIGURE 1:**
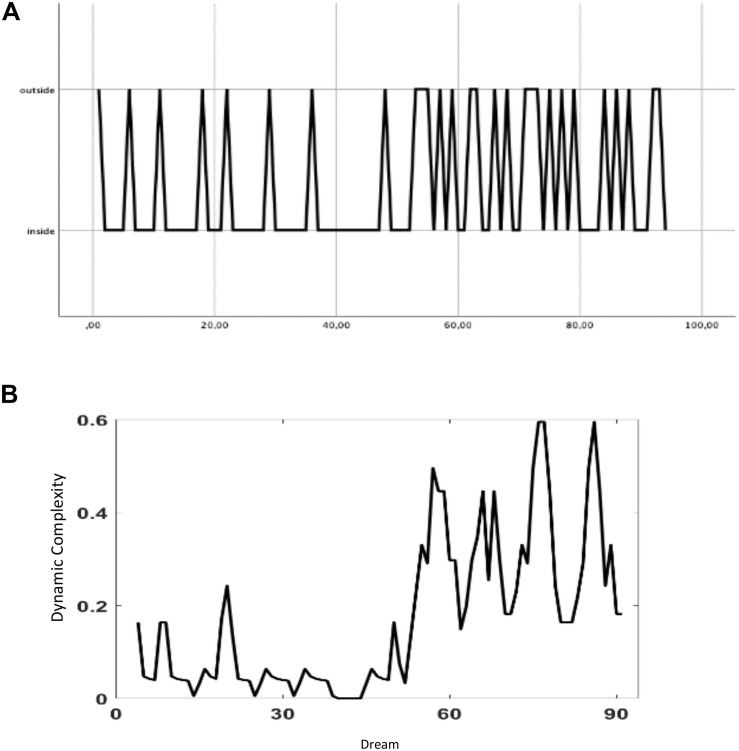
**(A)** Binary coded time series of dreams within the centroid (value 1) or outside the centroid (value 2) of the 10-dimensional factor space. **(B)** Dynamic complexity of the binary coded time series **(A)** (window width: 7).

As highlighted in the section “Materials and Methods,” the affective charge of dream narratives was calculated in terms of ED. The ED from the origin of the phase space and each dream vector was computed, and according to the overall mean of the EDs (arithmetic average = 0.536), the centroid was defined. Sixty-six dreams (70.2%) were proven to lie within the centroid and 28 dreams (29.8%) outside of it. Figure 1A shows their distribution according to the calculated centroid over the course of the psychotherapy. As can be seen, the frequency of highly affective-charged dreams (i.e., lying outside of the centroid) increases in the second part of the therapy. The DC of this binary oscillation is spontaneously increased after about the 58th dream (Figure 1B).

In order to estimate the probability that the frequency of affective-charged dream narratives (high vs low affective charge according to the calculated centroid) increases a function of time, a binary logistic regression has been performed. The logistic binary regression model identified the significant role of “time” (the sequence of dreams from 1 to 95) as a predictor for the probability of dreams to exceed the centroid threshold, that is, to be more associated to affective-laden meanings (Table 1). The predictor “time” was statistically significant (χ^2^ = 5.07, *df* = 1, *p* = 0.024). The concordant association of predicted probabilities and observed responses was 70.20%. Based on the odds ratio (ORs = 1.019), the logistic binary regression model reveals that the number of highly affective charged dream narratives increases during the course of the psychotherapy process.

**TABLE 1:** Logistic regression model with “time” as a predictor of the probability of dreams to overcome the centroid threshold.

	**β**	**SE**	**Wald**	***p***	**OR**
Time	0.190	0.009	4.758	0.029	1.019
Constant	−1.829	0.524	12.195	0.000	0.161

β, *regression weight of “time”; SE, standard error; Wald, test the significance of the relation between the independent variable and the criterion in the logistic model; *p*, probability of the Wald test; OR, odds ratio.*

### Hypothesis 2

**FIGURE 2:**
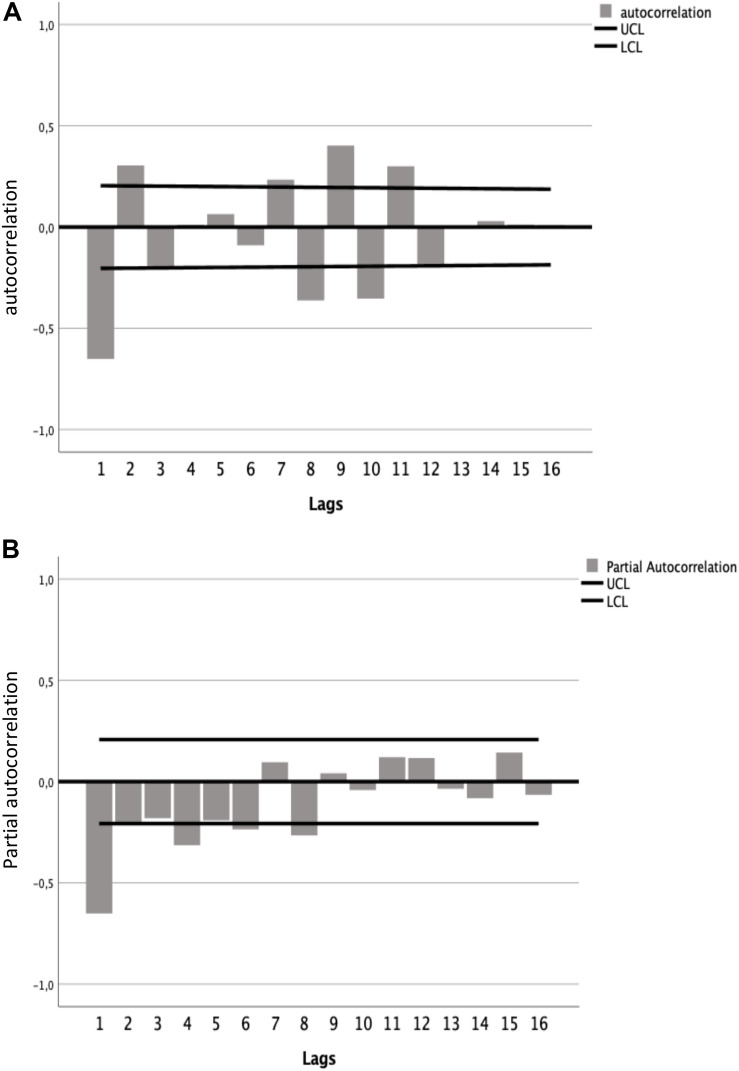
**(A)** Autocorrelation function (ACF) and **(B)** partial autocorrelation function (PACF) graphs. The straight lines above and below the 0.0 line indicate the 95% confidence interval (CI). ACF and PACF values (black bars) which clearly exceed the CI line appear only at lag 1, suggesting an AR1 (*p* = 1) and MA1 (*q* = 1) model.

HP2 was tested by means of an ARIMA model having the extent of the EDs as the dependent variable and time as the independent variable. On the basis of a preliminary estimation of the autocorrelation function (ACF) and the partial ACF (PACF) applied to the ED time series (Figure 2), the following parameters were set in the ARIMA_(_*_*p*_*,*_*d*_*,*_*q*_*_)_ model: *p* = 1, *d* = 1, and *q* = 1.

**FIGURE 3:**
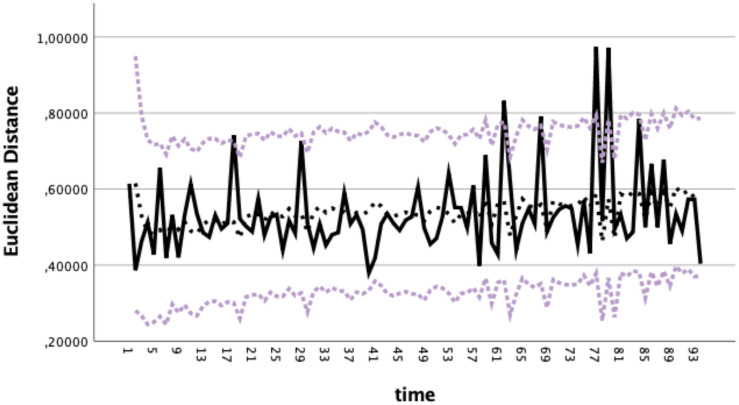
Time series of the Euclidean distances (EDs) of each dream from the origin in the factor phase space (black line) and the curve of the fitted ARIMA_(1,1,1)_ model (black dotted line) within a confidence band (dotted gray lines: upper and lower limits of the 99% confidence interval).

The ARIMA_(1_,_1_,_1)_ model fitted to the data (Figure 3) and explained a variance (*R*^2^) of.594, the Ljung–Box test (*Q* = 23.15; *df* = 16; *p* = 0.110) testing the white noise of residuals showed that the null hypothesis that all correlations are equal to zero cannot be rejected (where *p* > 0.05; thus, residuals represent white noise).

**FIGURE 4:**
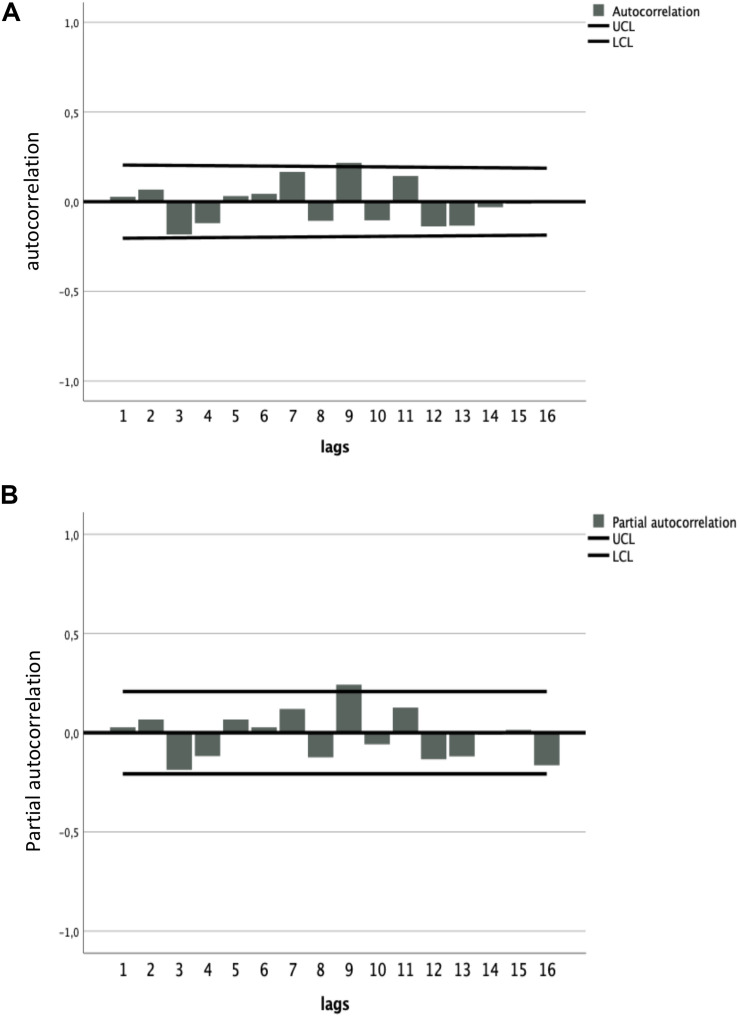
**(A)** Autocorrelation function (ACF) and **(B)** partial autocorrelation function (PACF) of the residuals of the ARIMA_(1,1,1)_ model. The straight lines above and below the 0.0 line indicate the 95% confidence interval (CI). The correlation values (gray bars) lie within the 95% CI limits, which indicates that the errors of the residuals are white noise. This proves that the model is appropriate for prediction.

The parameters of the ARIMA_(1_,_1_,_1)_ model AR1 and MA1 were statistically significant (*p* < 0.05 and *p* < 0.001, respectively; see Table 2). The ACF and PACF graphs (Figure 4) of the residuals of the ARIMA_(1_,_1_,_1)_ model confirmed that the data were fully modeled and that “time” predicts the extent of ED.

**TABLE 2:** The parameters of the ARIMA_(1,1,1)_ model.

	**Value**	**SE**	***t***	***p***
Constant	0.011	0.000	2.578	0.012
AR lag 1	–0.240	0.106	–2.272	0.025
Difference	1	–	–	–
MA lag 1	0.995	0.176	5.654	0.000

*SE, standard error; *t*, *t* value; *p*, probability of the *t* value.*

### Hypothesis 3

**FIGURE 5:**
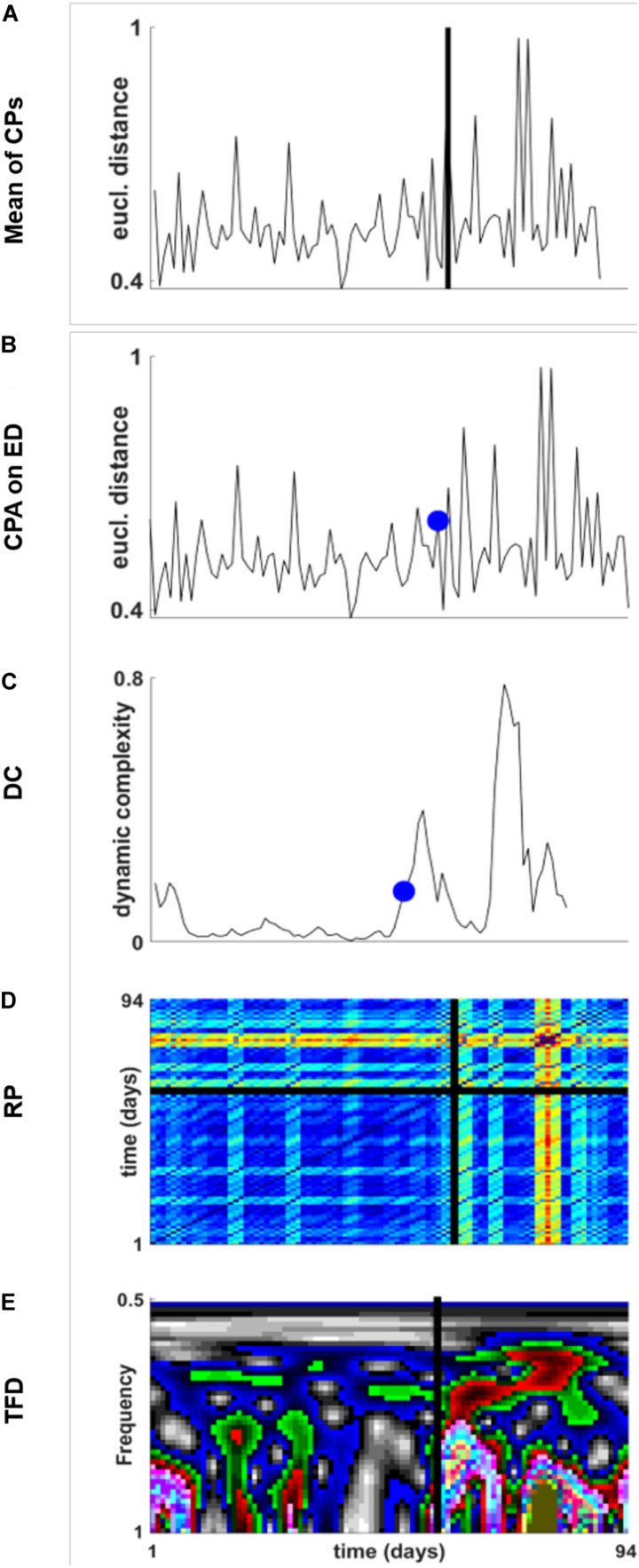
**(A)** Time series of the Euclidean distances of each dream (compare with the black curve in Figure 3). The straight vertical line indicates the average of the change points identified on the ED time series by CPA. Criterion of the CPA: changing variance. **(B)** CPA (blue dot) applied to the ED time series. **(C)** CPA (blue dot) applied to the DC (window width: 7) of the ED time series. **(D)** CPA (black vertical line) applied to the RP of the ED time series. Parameters: Three embedding dimensions and *τ* = 1. CPA was applied to each line and column of the RP. **(E)** CPA (black vertical line) applied to the TFD of the ED time series. The red colors indicate the highest amplitudes of the time-dependent frequency distribution. The average of the change points is at dream 58 [black line in panel **(A)**].

With reference to the ED time series of all dreams (Figure 3, black line), DC, recurrence, and frequency measures were applied (Figure 5). DC, RP, and TFD show a changed pattern after about two thirds of the dream sequence, with increased DC (Figure 5C), increased complexity in the sense of more transient and less recurrent patterns in the RP (Figure 5D), and increased frequency amplitudes (Figure 5E). In the TFD diagram, the emergence of the red amplitudes indicates the highest amplitudes of the frequency distribution. CPA was applied to the ED time series and to the DC, the RP, and the TFD of the ED time series. The change points (criterion: changing variance) indicated a transition at dream 57 in the ED time series (Figure 5B), at dream 60 in the DC of the ED time series (Figure 5C), at dream 59 in the RP of ED time series (Figure 5D), and at dream 57 in the TFD of the ED time series (Figure 5E). The average of these change points demarcates a pattern transition at dream 58 (straight line in Figure 5A).

## Discussion

The sequence of 95 dream narratives which were reported during a psychoanalytic therapy of more than 500 sessions realized over a period of more than 4 years—the famous case of “Amalie X”—revealed interesting results. The present work assumes dreams as a context which sustains the re-elaboration of relational meanings regulating the patient’s internal and external worlds. According to this framework, the ACASM was applied to the dreams × lemmas matrix obtaining a 10-dimensional factor space. Each factor represents an unconscious dimension which is based on strongly associated (co-occurring) words in the dream narratives.

According to our first hypothesis, the results highlighted an increased access to affective-laden meanings during the course of the psychotherapy. Concerning the second hypothesis, the results showed that the affective polarization of the dream narratives has been proven to increase following a non linear trend.

Taken together, the results shed light on the nature of the psychoanalysis as an intersubjective process aimed to sustain the patient toward a higher ability to get in touch with affective-laden meanings (i.e., unconscious dimensions) regulating and organizing life experience.

The dream narratives increase their frequency (HP1) in reaching highly affective-laden meanings, and the affective charges of the dream narratives increase in intensity among time (HP2). Taken together, HP1 and HP2 support the view of the clinical process as a recursive dynamics enriching the patient’s ability to explore—increasingly deep—unconscious dimensions (i.e., affective-laden meanings) in order to promote their cognitive–affective elaboration.

The third hypothesis is corroborated by showing a sudden jump of complexity offering a deeper understanding of the clinical process in terms of a non linear phase transition. Taken together, the converging evidence of DC, RP, and TFD of the ED dynamics, around dream number 58, a sudden change in the variability and complexity of the clinical process has been highlighted. The method of CPA following the criterion of changing variance was used to identify the transition in the measures and in the ED time series itself.

Hypothesis 3 enriches the view of hypotheses 1 and 2 on the clinical process: synergetics states that during psychotherapeutic processes, order parameters emerge which enslave the mental states of the patient and therapist. In consequence, the narratives of the dreams, the experienced scenes of the dreams, and the self-related and interactive process of psychotherapy get synchronized.

The phase transition highlighted in the 58th dream narrative—corresponding to the 328th psychoanalytic session—has been highlighted as a clinical turning point by several authors: the chronically depressed female patient who always avoided any close relationship, especially sexual contacts with men, and suffered from a reduced self-esteem and a distorted body image actively started to get in contact with men and had her first erotic and sexual experiences. Additionally, the patient emancipated from her mother with whom she experienced a very close, dependent, and symbiotic relationship. At the time of the phase transition, Thomä and Kächele (2006, p. 283, Figure 6.1) also reported on a discontinuous reduction of her verbal activity during the sessions. With reference to the five types of dream patterns identified by Roesler (2018a, 2020), Widmer (2019) visualized a frequency transition reflecting an increased ego strength of the patient and an intensified “authorship” of the dream ego.

The open question is if the phase transition after dream number 58 is relevant for the personality development of the patient. How is the relationship between the content(s) of the dreams, the ongoing psychotherapy, the development of the personality, the symbols and the themes, and the inner process (i.e., the core conflictual themes) related to the phase transition? Is it possible to find this phase transition also in the transcripts of the sessions? These questions will be investigated in the next step of the analysis of Amelie’s dream reports according to the transcripts of the therapeutic sessions.

## Conclusion

Taken together, the present results sustain the empirical effort of investigating the process of psychoanalysis by looking at the evolution of dreams. Nevertheless, some limitations that could direct future research efforts should be mentioned.

The study focuses exclusively on dreams without taking other reports or measures of the psychoanalytic process into account. In a next step, the transcripts of the therapeutic conversation should be analyzed in parallel to the dreams. For this case of “Amelie X,” the transcripts of 517 sessions are available. Secondly, in order to get a deeper understanding of therapeutic change processes, further data sources and parameters (e.g., self-assessments by electronic real-time monitoring devices, coding of session-by-session video tapes, and physiological measures during or in between sessions) should be used for a multilevel analysis of much more than one patient. Thirdly, the synergetic approach which was adopted here has a *post hoc* nature without any predictive value. Nevertheless, it is interesting to notice that the detected change point at about dream 58 corresponds to a clinical shift detected by previous clinical work.

Future research should take these limitations into consideration in order to relate qualitative changes of the dream dynamics to the therapeutic conversation and to the patient’s everyday life (see Thomä and Kächele, 2006). Albeit dreams are acknowledged as the “royal road” to the unconscious (Freud, 1900), the investigation of unconscious processes should be supplemented by the investigation of conscious processes, physiology, and behavior. Reaching such aim requires an interdisciplinary research paradigm able to describe the clinical process in terms of the interaction between the client’s lifestyle scenario, the therapeutic system, and the context of therapy (Schiepek et al., 1992). The present work underlines the importance of moving from the traditional outcome research toward a research on the process (Salvatore et al., 2009), including the measurement of the non linearly interrelated factors which are responsible for conveying change in a clinical case (Tschacher and Ramseyer, 2009; Haken and Schiepek, 2010; Schiepek et al., 2014, 2017; Schöller et al., 2018). The results and the adopted methodological framework are part of a general paradigm describing the clinical change dynamics in terms of self-organization and non linear systems theory.

## Data Availability Statement

The datasets generated for this study are available on request to the corresponding author.

## Author Contributions

All authors listed have made a substantial, direct and intellectual contribution to the work, and approved it for publication.

## Conflict of Interest

The authors declare that the research was conducted in the absence of any commercial or financial relationships that could be construed as a potential conflict of interest.

## Figures and Tables

**TABLE A1:** Description of factor 1 and factor 2 concerning the first 20 lemmas retrieved by dream narrative analysis.

**Factor 1**		**Factor 2**	
	
**Lemma**	**Squared cosine**	**Lemma**	**Squared cosine**
to see	0.742	root	0.969
uncle	0.681	tree	0.968
to find	0.571	to fall	0.594
Home	0.351	to want	0.593
to think	0.251	long	0.593
Father	0.229	figure	0.560
Image	0.135	relationship	0.481
remember	0.066	down	0.468
Dream	0.063	garden	0.420
to fall	0.048	to define	0.400
to bring out	0.043	speak	0.353
atmosphere	0.038	things	0.342
shoe	0.037	wait	0.331
dining room	0.033	ground	0.312
family	0.031	to wake up	0.188
			

**TABLE A2:** Squared cosine of each dream for the retrieved factors and calculated ED.

**Dream**	**Squared cosine in factor 1**	**Squared cosine in factor 2**	**ED**	**Position to the centroid**
10	0.021	0.113	0.114	Outside
21	0.074	0.034	0.082	Outside
26	0.037	0.030	0.047	Inside
42	0.004	0.083	0.083	Outside
53	0.009	0.064	0.065	Inside
60	0.000	0.047	0.047	Inside
64	0.022	0.117	0.119	Outside
82	0.039	0.001	0.039	Inside
86	0.004	0.096	0.096	Outside
92	0.035	0.010	0.036	Inside

**FIGURE A1:**
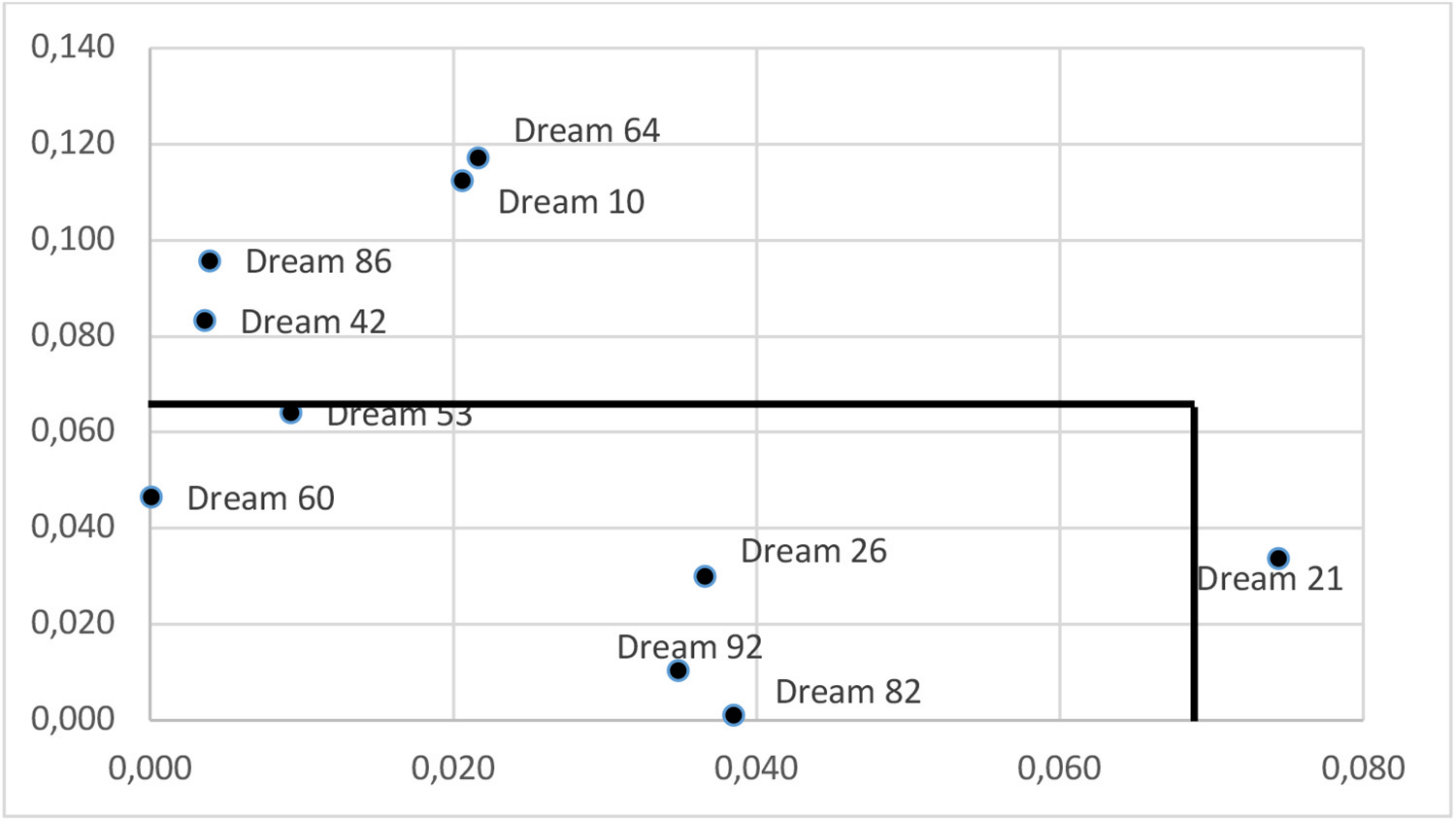
Representation of a sample of dreams related to the mean of the EDs (centroid). The *x*- and *y*-axis represent factor 1 and factor 2, respectively. The black line represents the centroid.

## Footnotes

1 ARIMA, which stands for autoregressive integrated moving average, is a model for time series data that incorporates both autoregressive and moving average features, along with detrending of the data. The AR part—*p* parameter—means that the values are regressed on their own lagged values; the MA part—*q* parameter—means that the regression error is a linear combination of past error terms; thus, it requires a non linear estimation algorithm to be used. Finally, the I part—*d* parameter—means that the data have been differenced to remove trend. If on one hand, ARIMA models could be assumed as a linear model (in that the parameters are linear), strictly speaking, they could not be thought of that way: linear models generally involve a dependent variable that is regressed on a number of independent variables, rather than the same variable at previous time points. In our case, the presence of the MA component let us consider the ARIMA as a non linear model.

## Appendix

In the following, we present an example to highlight the factors retrieved by MCA, their interpretation, their EDs, and their categorization as lying inside or outside the centroid. In this illustrative example, the analysis reveals two factors, and the factor interpretation is based on 20 lemmas characterizing the factors. This result is able to restore the conceptual and clinical value of the adopted measures.

Table A1 offers an example concerning the characterization of two factors according to 20 retrieved lemmas detected by the MCA procedure. Based on the squared cosine which represents the degree of association of each word to the retrieved factors, the factors have been interpreted as “family images” (factor 1) and “social actions” (factor 2). We remind that here we are reporting just an example of the performed analysis in order to shed light on its rationale; as a matter of fact, the paper is focused on formal and quantitative aspects aimed to present technical and formal solutions of analysis able to track dream evolution among the clinical process; thus, it does not take into consideration the qualitative aspects—i.e., the meaning of retrieved factors. Further studies will investigate the contents of factors, dreams, and their clinical evolution.

In Table A2, we report the squared cosine of 10 selected dreams out of 95 concerning factor 1 and factor 2 together with the calculated ED from the origin of the phase space defined by factor 1 and factor 2, respectively. The ED from the origin of the factorial space was calculated for each dream as $\sqrt{{\left(\mathrm{squared}\,\mathrm{cosine}\,\mathrm{factor1}\right)}^{2}-{\left(\mathrm{squared}\,\mathrm{cosine}\,\mathrm{factor2}\right)}^{2}}$. The centroid, calculated as the arithmetic mean of the EDs of the 95 dream narratives according to factor 1 and factor 2, was 0.67.

In Figure A1, we represent graphically the factorial space defined by factor 1 and factor 2 and the calculated centroid. The higher the ED value for each dream, the greater the incidence of one or more factors in determining the affective-laden meaning of a dream.
